# Educational intervention and livestock ownership successfully improved the intake of animal source foods in 6–23 months old children in rural communities of Northern Ethiopia: Quasi-experimental study

**DOI:** 10.1371/journal.pone.0277240

**Published:** 2022-11-04

**Authors:** Mekonnen Haileselassie, Getachew Redae, Gebretsadik Berhe, Carol J. Henry, Michael T. Nickerson, Afework Mulugeta

**Affiliations:** 1 School of Public Health, College of Health Sciences, Mekelle University, Mekelle, Ethiopia; 2 Tigray National Regional State, Bureau of Science and Technology, Mekelle, Tigray, Ethiopia; 3 College of Pharmacy and Nutrition, University of Saskatchewan, Saskatoon, Canada; 4 Department of Food and Bioproduct Sciences, University of Saskatchewan, Saskatoon, Canada; University of North Carolina at Chapel Hill Gillings School of Global Public Health, UNITED STATES

## Abstract

**Background:**

Animal source foods (ASFs) are rich in high-quality proteins, including essential amino acids and highly bioavailable micronutrients vital for child growth and cognitive development. But, the daily consumption of ASFs among 6–23 months old children is very low in Tigray, Northern Ethiopia.

**Objective:**

The study aimed to assess the effectiveness of nutrition education intervention to improve the consumption of ASFs among 6–23 months old children from rural communities with strict religious fasting traditions of avoiding intake of ASFs in Northern Ethiopia.

**Methods:**

A quasi-experimental study was conducted in two food insecure districts namely Samre Seharti (intervention) and Tanqua Abergele (comparison). The mother-child pairs in the intervention group (n = 140) received nutrition education based on the barriers and available resources for optimal consumption of ASFs among children and followed up for nine months. The mother-child pairs in the comparison group (n = 153) received routine nutrition education. The data were collected using a pre-tested structured questionnaire. The baseline and endline data assessment included interviews on socio-demographic and socio-economic status, dietary intake, and child feeding practices. The effectiveness of the intervention was measured using the difference-in-difference (DID) analysis model.

**Results:**

At endline, the consumption of ASFs among children was 19.5 percentage points higher in the intervention group compared with the comparison group (p = 0.008). In addition, there was a significant increase in egg consumption among children in the intervention group (DID of 16.9, p = 0.012) from the comparison group. No child was consuming meat at baseline in both the intervention and comparison arms and it was very low at endline (5.2% vs. 7.9%). Overall, the proportion of children that consumed eggs in the intervention group was higher than in the comparison group in households that owned sheep and goats (4.8% vs. 21.4%, p = 0.050) and chicken (6.3% vs. 43.8%, p = 0.002) after education interventions. However, no statistically significant difference was observed between cow ownership and milk consumption among children (p>0.05).

**Conclusions:**

Age-appropriate educational interventions for mothers and owning small livestock in the household can improve the consumption of ASFs and eventually the minimum diet diversity of children in communities with strict religious traditions of avoiding ASFs during the fasting seasons.

## Introduction

Although Ethiopia has made remarkable progress against under-nutrition, indicators of child nutrition remain extremely poor across the country. Children under-five remain stunted (36.8%) and wasted (7.2%). And this varies significantly by region with 48.7% of under-five children stunted and 9.2% wasted in Tigray [1]. These high rates of under-nutrition are caused by many factors, most of which relate to poor diet or severe and repeated infections [2]. One neglected dimension of Ethiopian’s child under-nutrition problem is low animal source foods (ASFs) intake.

Animal source foods including meats, eggs, and dairy products are rich in high-quality proteins and have highly bioavailable micronutrients essential for child growth and cognitive development [3, 4]. Adequate intake of ASFs was strongly associated with better growth and mental development, improved activity, school performance, less morbidity, and anaemia, as well as higher immune function in young children [5, 6]. An interventional study in a rural area of Malaysia reported that consuming ASFs was a significant factor in increasing the height, weight, and Mid Upper Arm Circumference (MUAC) of children [6]. A systematic review by Dror et al. (2011) also reported that consuming ASFs is linked to improved cognitive function among malnourished children in low-income settings [7].

However, the consumption of ASFs among infants and young children in Ethiopia is very low [8–12] despite the largest livestock population in the country. In this region, as in many developing countries, the diets of children are predominantly cereal-based with low energy and nutrient density [9, 13]. According to the 2016 Ethiopian Demographic Health Survey (EDHS) report, only 8% of breastfed children aged 6–23 months consumed meat, 38.2% (dairy products), and only 17% reported egg consumption in the 24 hours prior to the interview [14]. Similarly, a study conducted in Tigray region reported that 1.1%, 24.9%, and 11.7% of children aged 6–23 months consumed meat, eggs and dairy products, respectively. As the result, only 7.2% of them meet the WHO recommended dietary diversity [13]; possibly because of the diets being extremely monotonous, which could be low consumption of ASFs among infants and young children.

Some reported factors that influence the consumption of ASFs among infants and young children in Ethiopia are cultural and religious reasons, availability, lack of nutrition knowledge, price, and economic status of the household [8, 15–17]. Hence, educational strategies focusing on increasing the utilization of ASFs to improve diet quality and diversity are needed, based on contextual factors that constrain the consumption of ASFs in vulnerable households.

Various studies demonstrated that nutrition education can improve mothers’ feeding and nutrition knowledge [18–20]. For example, in rural Ecuador, the intervention of one egg per day for 6–9 months old children was assigned to treatment for six months, and a comparison followed a usual diet. As a result, the length-for-age *Z* score increased by 0.63 and the weight-for-age *Z* score by 0.61 among the study children relative to the comparison group [21]. Likewise, observational analyses of a household survey in Rwanda showed that children from cow-owning households increased in HAZ measures by 0.55 to 1.13 standard deviations compared to families of children without cows [22]. In rural Nepal, children of nutrition-sensitive community-level program participant mothers were 1.4 times more likely to have consumed ASFs than the comparison group [23].

Similarly, in Ethiopia there are a bulk of interventional studies, which were conducted on community and facility-based interventions delivered through complementary feeding messages, training of mothers in groups, counseling and support at home level and facilitating participatory family discussion on appropriate IYCF behaviours and practices [24–28]. Most of these interventions were focusing on improving early initiation and exclusive breastfeeding practices, introduction of complementary foods, dietary diversity, and meal frequency thereby to improve IYCF practices [24–28]. As a result, these interventions had more impacts on dietary diversity, minimum meal frequency, and help mothers to adopt and sustain positive child feeding behaviours and practices. However, interventional studies are limited to improving ASFs consumption among infants and young children in the country.

Therefore, the present study was conducted to improve the consumption of ASFs in infants and young children through educational intervention for mothers based on contextual factors that constrain the consumption of ASFs from food insecure districts where ASFs are avoided during the religious fasting season. The intervention districts are project areas for the Seqota Declaration interventions with a target of achieving an end to childhood stunting by 2030 [29]. The Seqota Declaration is a government implementation plan (2016–2030) that leverages pre-existing policies, strategies and programs in place to make the best use of lessons learned and apply best practices at scale in a targeted approach. It is expected that through the effective roll-out of this implementation plan, Ethiopia will experience a paradigm shift towards eliminating child undernutrition by 2030. Hence, the present study results could be used as an input to support the implementation of the Seqota Declaration.

## Methods and materials

### Study setting

The study was conducted in two districts of Tigray, Ethiopia situated along the Tekeze river basin, namely Samre Seharti and Tanqua Abergele districts, with a total population of 144,527 and 120,180, respectively [30]. There are 13,054 children of 6–23 months old in both districts. The study groups were made from two districts to prevent information contamination between intervention and comparison groups. These two districts were purposively selected because these are project areas for the Seqota Declaration interventions with a target of achieving an end to childhood stunting by 2030 [29], which calls for scaling up and intensification of the high-impact nutrition interventions. The two districts also had a high prevalence of child stunting (above 40%) [31] and a large livestock population compared to others [30]. The livelihood of the study communities depends on agriculture (crop and livestock production). The most common livestock include cattle, sheep, goats, and chickens. Livestock products (e.g., butter, eggs) and other economic services from the livestock (e.g. goats, sheep, and chicken) are often seen as sources of cash in times of financial constraints. The communities are largely Orthodox Christians, where the consumption of ASFs during fasting occasions is strictly prohibited.

### Study design

A quasi-experimental design on the same cohort using before and after surveys in the intervention and comparison districts was used to evaluate the effectiveness of nutrition education intervention on ASFs consumption among infants and young children from October 2019 to June 2020 in Northern Ethiopia. For logistic reasons, we have chosen to allocate Samre Saharti to the intervention and Tanqu Abergelle to comparison groups purposively instead of randomizing individuals from each district.

#### Inclusion criteria

Breastfeeding infants aged six to eight months; parents (or mothers), who are permanent residents of the study communities and have no plans to move away during the intervention period. Breastfeeding children were selected because continued breastfeeding rates at two years were high (92.4%) at the regional level [14]. And six to eight months age children were included, because the consumption level of ASFs among them was extremely low in the study area [32].

#### Exclusion criteria

Infants with an obvious health problem and impaired feeding or physical growth measurements; children who were twins; mothers who refused to participate; parents (or mothers) with no plans to stay in the communities during the intervention period.

### Sampling technique and sample size calculation

**Fig 1:**
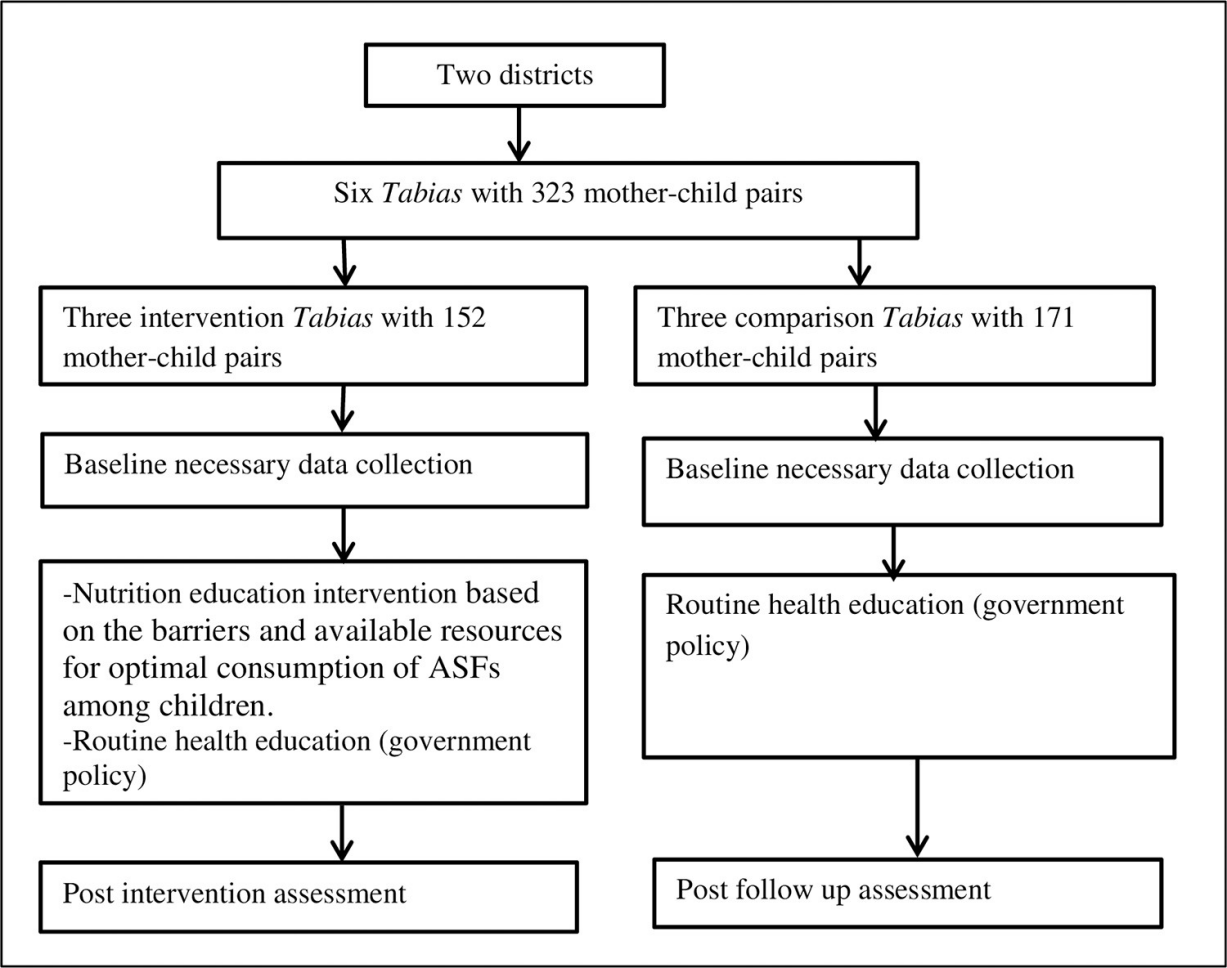
Schematic representation of the intervention protocol.

After knowing the total children of 6–23 months age in the two districts from the health post registry, for logistic reasons, only three Tabias from the intervention and three Tabias from the comparison district were included using a simple random sampling technique “Fig 1”. There are 43 Tabias in both districts. Tabia is the lowest administrative unit consisting of about 5,000 people [33]. Both districts had similar background characteristics such as geographical location, cropping pattern, near-to-market access, socio-economic status, feeding system and habit, child health services, baseline child health and nutritional status and livestock population. Finally, mothers with six to eight months of age children were identified and listed. Then from the list of mothers, study participants were selected using a systematic random sampling method as stated below.

There were 1531 and 1224 mothers with 6–23 months old children in Samre Saharti and Tanqu Abergelle districts, respectively. From those, the total numbers of six to eight months of age children were extracted. And once they were known in the study Tabias of each district, the sampling interval (K) was determined by dividing the number of units in the population by the desired sample size (N/n). Then selecting a number between one and K at random called the random start, and every K^th^ unit was selected after that first number was known to get the total samples “Table 1”.

**Table 1:** Total number of samples from each study Tabia.

Samre Saharti (district)			Tanqu Abergelle (district)		
Tabias	Total number of mother–child pairs (6-23m)	Sampled (6-8m)	Tabias	Total number of mother–child pairs (6-23m)	Sampled (6-8m)
Adikaela	593	66	Jijke	362	51
MariamMoko	502	56	Simret	507	70
Adishishay	436	49	Tekleweini	355	50

m = month

The sample size was determined based on the assumptions of α = 0.05, β = 80%, and a two-tailed test to detect a difference of 15 percentage points [34]. The consumption of a diet composed of egg among breastfed children aged 6–23 months were reported 17% [14]. We chose this indicator to inform the sample size because as many households in the rural areas own poultry. In addition, a 10% contingency for loss to follow-up was considered [35]. To adjust for clustering at the Tabia level, we used a design effect (1.5) which was calculated by considering the intra-class correlation (roh) = 0.01 and coefficient of variation (CV) = 0.086 [36].

Based on these parameters, 171 (for intervention) and 171 participants (for comparison) were included in the study area. But at the start of the intervention, 152 and 171 participants from the intervention and comparison arms, respectively consented to enroll in the study.

### Intervention strategies

**Fig 2:**
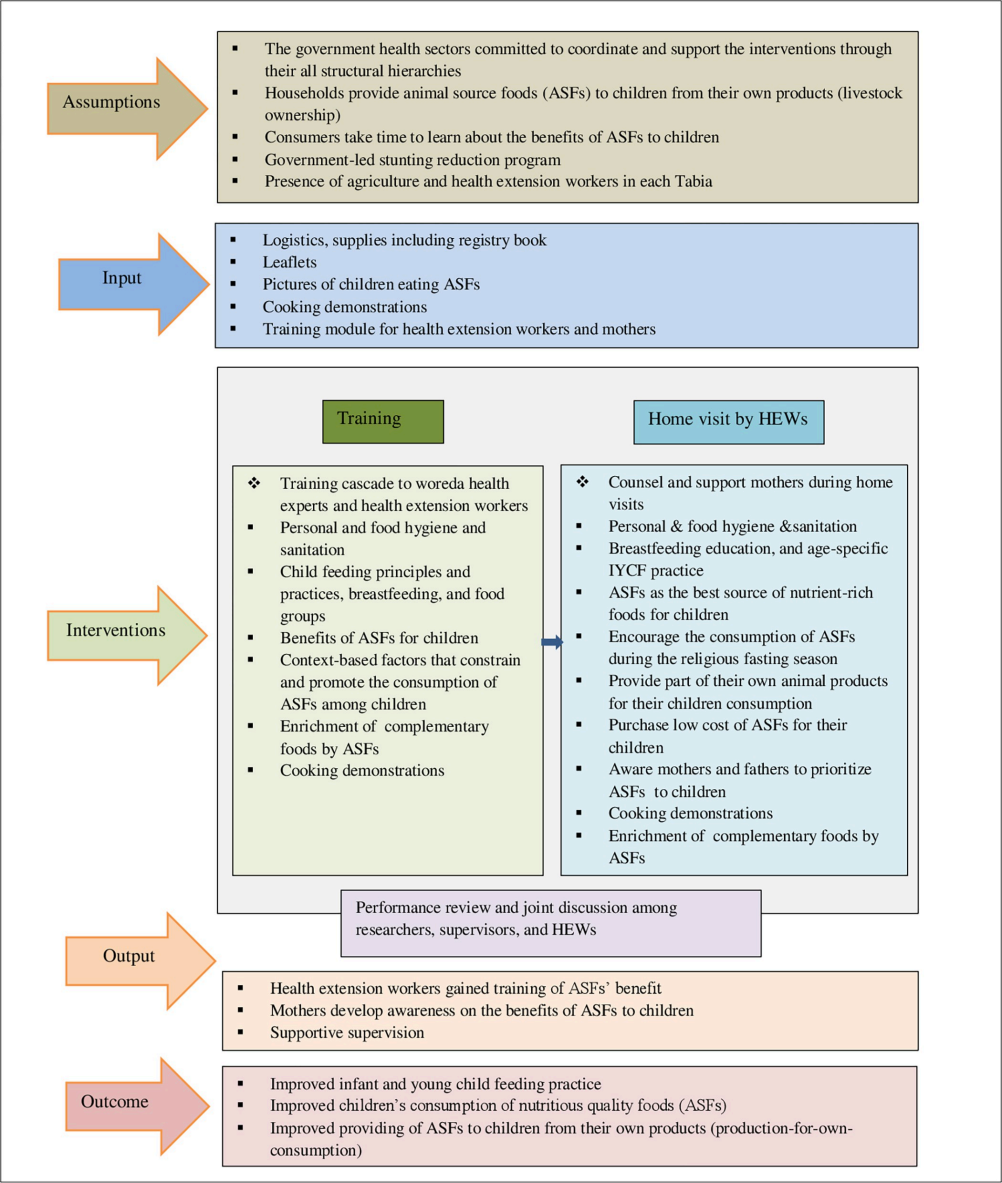
Logic framework of nutrition education interventions for optimizing the consumption of ASFs among young children in selected districts of Northern Ethiopia.

An interventional study was designed to assess the effectiveness of community-based nutrition education in improving the consumption of ASFs among 6–23 months old children in the study area. Based on the previously assessed barriers and available resources for optimal consumption of ASFs among 6–23 months old children in the study area [8, 32], it was invited to form the basis of a logic framework for a multifaceted intervention. It was postulated that community engagement would increase the low consumption of ASFs among young children through improving the awareness of health extension workers and mothers in the study area “Fig 2”. Nutrition education was given to mothers every two weeks of home visits. The nutrition education was conducted by trained Health Extension Workers (HEWs). Similarly, training and supervision of HEWs was carried out by the research team and woreda health workers “S1 Table”.

### Training of health extension workers

To reinforce and support mothers’ behavioral change, the HEWs were trained on the benefits of the consumption of ASFs for infants and young children and the need for regular and scheduled home visits to target households. The training of the HEWs was made to focus on action-oriented knowledge and skills to effectively counsel and negotiate with mothers to adopt recommended practices. In each intervention Tabia, two HEWs were recruited and trained to counsel and support mothers during home visits; and mobilize mothers for outcome assessments. All recruited HEWs and their supervisors from the health center and agriculture extension workers were centrally trained for two days. It was focused on orientation and introduction, about child under-nutrition, why nutrition matters during the first two years of life, feeding principles and practices, use of ASFs for children, feeding frequency, and food groups. Based on the previous qualitative findings on the barriers and facilitators of ASFs consumption among infants and young children in the study area [8], training and educational resources were developed to support the implementation of the intervention. Health extension workers also focused on home visit tasks and procedures, use of counselling resources (such as pictures and leaflets), supervisory visits, and training evaluation. Each HEW was assigned 25 mothers residing in her Tabia, and each mother was individually visited and counselled every two weeks for nine months of the intervention period. Mothers with sick children or children with feeding problems were offered additional visits.

### Supervision of health extension workers

Supervisory visits coupled with observed counselling sessions, review of home visits and discussions on feedback were made to assess the quality of the counseling services and to address any challenges faced during the intervention period. An observational checklist was used to assess practice on effective use of counselling resources, offer referrals appropriately, promote as ASFs are the best source of nutrient-rich foods for children aged 6–23 months among mothers, and discuss practical solutions for any problem they faced “S1 Table”. Supervisory visits and discussions among the research team, supervisors, and HEWs took place every month, and phone communication was done at least weekly.

### Education of mothers

Nutrition education, mainly on ASFs and counselling was conducted with mothers of recruited infants and young children in the study area. The education focused on age-specific feeding recommendations made by WHO [8], and the effectiveness of a nutrition education package in improving feeding practices (ASFs consumption) and dietary adequacy [37] with the same women as described below “Table 2”.

**Table 2:** Contents of the nutrition education sessions provided to mothers of 6–23 months old children.

Contents
1. Contents related to perceived barriers for ASFs use and appropriate complementary feeding practices: • Discuss the benefits of appropriate complementary feeding practices and of ASFs use in complementary feeding practices (e.g., a child with good nutritional status is very active, strong, and healthy); ASFs have good nutritional benefits (such as protein, iron, zinc, calcium, vitamin A, vitamin B12) for child growth and mental development) • Cook, mash, and feed ASFs (e.g. eggs, beef, mutton, chicken) • Provide whole milk and undiluted milk rather than the skim milk • Cook and boil rather than serving in the raw form • Keep ASFs in a hygienic way to prevent any zoonotic and foodborne diseases • During the selling of ASFs, advise mothers to purchase other food groups for children (eg. Fruits and vegetables, less price animal products) • Priority is given to infants and young children rather than others (fathers) • Discuss the beliefs and norms that affect the consumption of ASFs among children and mothers should encourage the consumption of ASFs during the religious fasting season • Discuss the role of ASFs in promoting growth and good health, and cereal-based complementary foods have incomplete protein and micronutrients • Reduce the market-oriented production of ASFs and share part of the products for their child consumption • Enrich complementary foods by adding ASFs such as milk and egg while preparing porridge from a combination of cereal flours. • Feed the sick baby during and after illness (diversity, frequency, consistency, and amount). • Hygiene (safety, handling & storage of food), home and environment sanitation • Allocate a separate feeding plate to quantify food for the child 2. Perceived opportunities for ASFs use and appropriate complementary feeding practices (e.g., availability of nutrition experts, cooking demonstrations, livestock ownership) • Providing training to the nutrition experts to promote the consumption of ASFs among infants and young children • Cooking demonstrations and picture-based training on the importance of ASFs and child development • Counsel mothers to provide the ASFs from their own production (e.g. if they have cow milk, chicken, sheep, and/or goat, the mothers were counseled to provide milk, egg or chicken meat) • Enrichment of the complementary food with dry meat (quanta) powder by purchasing fresh meat from butchery shop

### Data collection

The data collection was held in two phases—beginning before the intervention and one month after the last educational sessions. First, interviews were conducted face to face with the mothers in the health center and health posts by trained data collectors using pre-tested, semi-structured questionnaires for assessing socio-demographic characteristics, socioeconomic status, and status of ASFs consumption as complementary feeding “S1 Questionnaire”. The questionnaire was adapted and designed from EDHS data [14], questionnaires for child health and nutrition, and WHO indicators for assessing infant and young child feeding practices measurement [38, 39], prior to the revised guidance of 2021 [40].

A 24-hour dietary recall method was used to assess dietary diversity in each household by asking the mothers about breastfeeding and listing all food consumed by each child in the 24-hours preceding the survey [39]. All the reported food items were classified into seven food groups: cereals, legumes and nuts, dairy products (milk and milk products), flesh foods (meat, chicken, and liver/organ meats), eggs, and vitamin A-rich fruits and vegetables, and other fruits and vegetables. Children receiving four or more food groups were categorized as meeting the minimum dietary diversity; or else, they were considered as getting low minimum dietary diversity [39].

### Data analysis

The quantitative data were entered into Epi data version 3.1 and exported to and analyzed using statistical software IBM SPSS Version 20 and Stata Version 8 SE. All continuous variables were checked for normality using the skewness and kurtosis test. The socio-economic status of each household was created by principal component analysis (PCA) using a set of items by stratification of the households into wealth quintiles [41]. The analysis was done using a set of items associated with house and land ownership, house construction materials, different types of durable assets, livestock ownership, and access to utilities. The first component derived from component scores was used to divide household socio-economic status into tertiles [41]. To create wealth tertiles, we grouped the asset index into nearly equal sized groups of households’ socioeconomic status based on the asset scores. And wealth score were created to categorize households as low, middle and high [42].

Baseline characteristics between intervention and comparison groups were compared using the t-test for continuous variables and chi-square tests for categorical variables. Difference-in-differences (DID) analysis was employed to evaluate the contribution of the intervention package to the children’s feeding practices. The difference-in-difference analysis is usually used to study the causal relationships in public health settings where carrying out randomized control trials may not be feasible or unethical [43]. Difference-in-difference/double differences analysis was based on comparing the percentage differences in the intervention district to differences in the comparison districts by assuming that trends in both groups were the same in the absence of the intervention.

ASF consumption of children in relation to livestock ownership was restricted to foods (meat, milk, and egg) derived from each livestock group (cow, sheep and goat, and chicken). Multicollinearity among the variables was detected using the variance inflation factor (VIF) showing that there was no multicollinearity (VIF < 10) [44]. Statistical significance was set at p-values < 0.05.

### Ethical consideration

The ethical review committee of Mekelle University, College of Health Sciences approved the study protocol with a reference number of ERC 1432/2018 and support letter was obtained from the Tigray Regional State Health Bureau. Written informed consent was obtained from mothers to proceed with the data collection process after they had been briefed on the purpose of the study, benefit and risk, confidentiality of the information and the voluntary nature of the participation in the study. Names and other personal identifiers were not recorded without their consent to maintain confidentiality. After the data collection, for the purpose of ethical issues, a message was provided for four hours to the comparison groups that the intervention group received during nutritional education periods.

## Results

### Socio-demographic characteristics

Table 3 presents the overall socio-demographic characteristics and economic status of study mother–child pairs in intervention and comparison groups. At baseline, 171 mothers with children 6–8 months of age were enrolled in the comparison and 152 in the intervention area. Nine months later, the endline survey included 140 mothers in the intervention and 153 in the comparison areas. Among the study mothers with children 6–8 months old, 61.5% and 64.1% never attended school, respectively, in the intervention and comparison areas. During the baseline, the mean age of study children was slightly higher in the intervention area (7.3 months) than in the comparison (7.1 months) area. Both surveys included slightly more boys than girls in the intervention and in the comparison area. During a difference-in-difference analysis, all the characteristics of children and families, and the feeding practices of infants and young children at baseline were comparable in both study areas. Chicken were the most common livestock asset in both the intervention and comparison areas.

**Table 3:** Socio-demographic and economic characteristics of participants at baseline in rural districts of Tigray, Ethiopia (N = 293).

Socio-demographic characteristics	Comparison, n(%)	Intervention, n(%)	p-value
Proportion of female children	73(47.7)	69(49.3)	0.737
Proportion of fathers without formal education	69(43.1)	58(41.4)	0.329
Proportion of mothers without formal education	98(64.1)	86(61.5)	0.376
Proportion of household livestock owners	134(87.6)	127(90.7)	0.71
Types of livestock owned			
Cow	37(24.2)	37(26.4)	0.936
Goat	42(27.5)	42(30)	0.630
Chicken	55(35.9)	48(34.3)	0.766
Socio-economic status			
Low	58(.37.9)	40(28.6)	0.069
Middle	43(28.1)	49(35.0)	0.532
High	52(34.0)	51(36.4)	0.922
Average family size of households (Mean ± SD)	5.53±4.282	5.63±1.847	0.808
Average age of child in months (Mean ± SD)	7. 1 ± 0.801	7. 26±0.836	0.840

### Consumption of animal source foods

**Fig 3:**
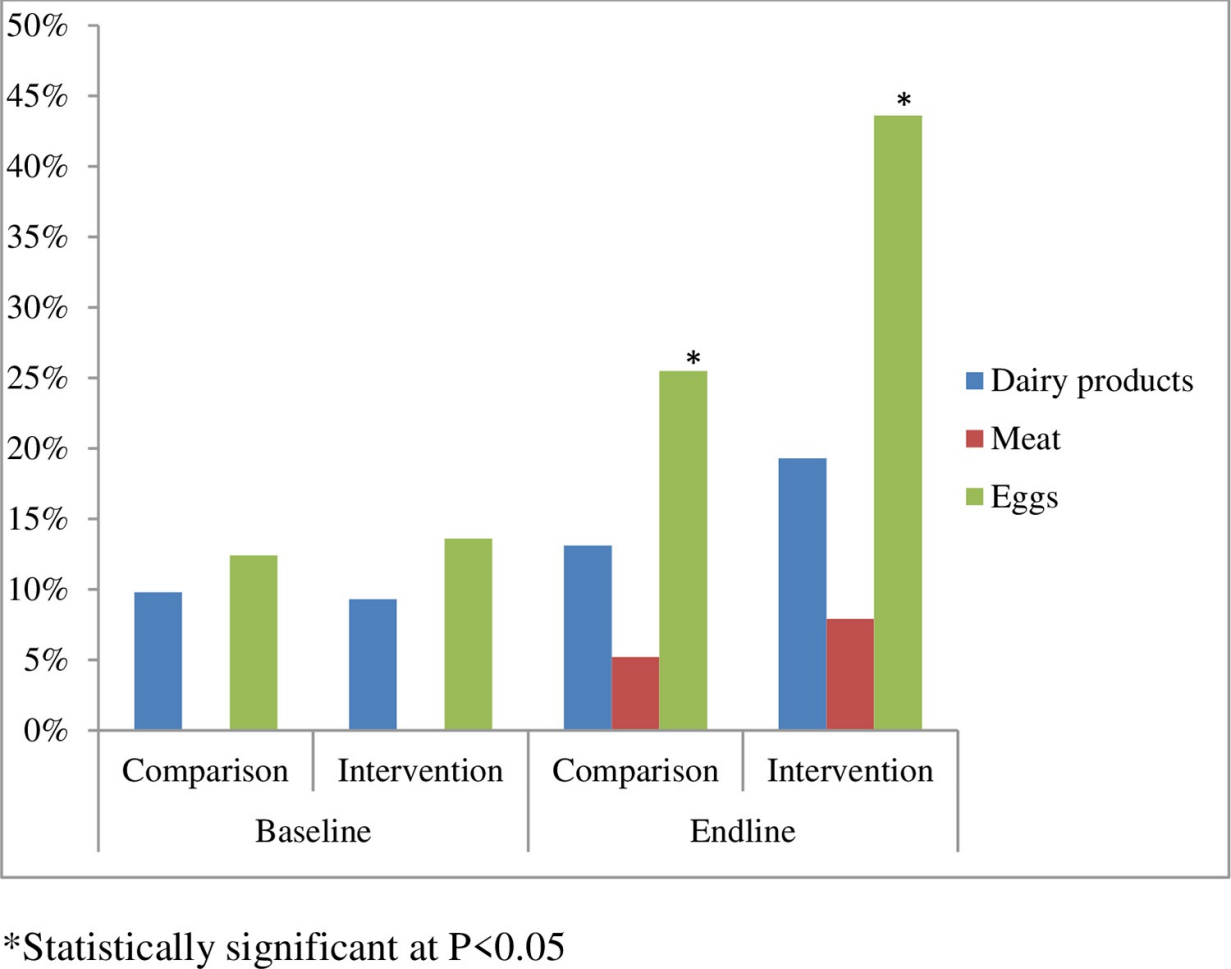
Percentage of overall study children who consumed ASFs in the intervention group compared to the comparison group over the fellow up period.

At baseline, the study groups were comparable regarding the consumption of the different food groups with no statistical difference among each Tabias of the study groups “S2 Table”. Table 4 presents results on child feeding practices in the intervention and comparison groups at baseline and endline. At baseline, the two groups were comparable regarding the consumption of ASFs with no statistical difference between the intervention and comparison groups. However, the intervention significantly influenced the consumption of eggs (p = 0.012). In the intervention area, consumption of eggs increased by 16.9 percentage points. After the nutrition education program in the intervention group, it was also significantly influenced the total consumption of ASFs among children (p = 0.008), which was increased by 19.5 percentage points. Both study arms, however, were observed to have no significant change in the frequency of meat (p = 0.363) and milk (p = 0.222) consumption “Fig 3”.

**Table 4:** Comparison of feeding practices between the intervention and comparison groups of children in rural districts of Tigray region, Ethiopia (difference-in-difference analysis).

Food groups	Baseline				Endline				Intervention effect (DID estimates)	p-value
	Comparison (n = 153)	Intervention (n = 140)	Diff in (%)	p-value	Comparison (n = 153)	Intervention (n = 140)	Diff in (%)	p-value		
Grains, roots and Tubers	128(83.7%)	117(83.6%)	-0.1	0.979	150(98%)	134(95.7%)	-2.3	0.493	-2.2	0.641
Legumes and nuts	57(37.3)	58(41.4%)	4.1	0.460	97(63.4%)	95(67.9%)	4.5	0.430	0.3	0.972
Dairy products	15(9.8%)	13(9.3%)	-0.5	0.894	20(13.1%)	27(19.3%)	6.2	0.111	6.7	0.222
Meat	0	0	0	1.00	8(5.2%)	11(7.9%)	2.6	0.198	2.6	0.363
Eggs	19(12.4%)	19(13.6%)	1.2	0.809	39(25.5%)	61(43.6%)	18.1	0.000	16.9	0.012
ASFs	27(17.6%)	27(19.3%)	1.6	0.751	54(35.3%)	79(56.4%)	21.1	0.000	19.5	0.008
Vitamin A-rich fruits and vegetables	4(2.6%)	6(4.3%)	1.7	0.591	17(11.1%)	19(13.6%)	2.5	0.429	0.8	0.858
Other fruits and vegetables	16(10.5%)	24(17.1%)	6.6	0.154	38(24.8%)	45(32.1%)	7.3	0.119	0.7	0.925
Minimum diet diversity	5(3.3%)	7(5.0%)	1.7	0.629	20(13.1%)	34(24.3%)	11.2	0.002	9.5	0.062
Minimum meal frequency	43(28.1%)	43(30.7%)	2.6	0.625	107(69.9%)	101(72.1%)	2.2	0.679	-0.4	0.958

DID, Difference-in-difference; ASFs, combined animal source foods (dairy products, meat, and eggs)

### Livestock ownership and ASFs consumption

During the baseline, livestock ownership didn’t significantly influence the consumption of ASFs among children in both the intervention and comparison areas “Table 5”. Sheep and goats’ ownership was considerably higher among children in the intervention than comparison group fed on milk (DID of 11.9, p = 0.283). The proportion of children that consumed eggs in the intervention group was higher than the comparison group in households that owned sheep and goats (DID of 26.2, p = 0.050) and chicken (DID of 37.5, p = 0.002) after education interventions. Although the association was not statistically significant between the two arms, meat consumption was slightly higher in all livestock owners of the intervention area during the endline.

**Table 5:** Associations between specific livestock ownership and animal source food consumption among children in rural districts of Tigray region, Ethiopia (difference-in-difference analysis).

Household livestock ownership	ASFs	Baseline				Endline				Intervention effect (DID estimates)	p-value
		Comparison	Intervention	Diff in (%)	p-value	Comparison	Intervention	Diff in (%)	p-value		
		n(%)	n(%)			n(%)	n(%)				
Cow ownership	Milk	2(5.4%)	3(8.1%)	2.7%	0.710	5(13.5%)	6(16.2%)	2.7%	0.710	0.0%	1.00
	Meat	0.0%	0.0%	0.0%	1.00	1(2.7%)	2(5.4%)	2.7%	0.411	2.7%	0.561
	Egg	1(2.7%)	3(8.1%)	5.4%	0.462	8(21.6%)	5(13.5%)	-8.1%	0.271	-13.5%	0.195
Sheep ownership	Milk	6(14.3%)	5(11.9%)	-2.4%	0.761	5(11.9%)	9 (21.4%)	9.5%	0.225	11.9%	0.283
	Meat	0.0%	0.0%	0.0	1.00	1(2.4%)	3(7.1%)	4.8%	0.152	4.8%	0.310
	Egg	8(19.0%)	6(14.3%)	-4.8%	0.612	11(26.2%)	20(47.6%)	21.4%	0.023	26.2%	0.050
Goat ownership	Milk	6(14.3%)	5(11.9%)	-2.4%	0.761	5(11.9%)	9 (21.4%)	9.5%	0.225	11.9%	0.283
	Meat	0.0%	0.0%	0.0%	1.00	1(2.4%)	3(7.1%)	4.8%	0.152	4.8%	0.310
	Egg	8(19.0%)	6(14.3%)	-4.8%	0.612	11(26.2%)	20(47.6%)	21.4%	0.023	26.2%	0.050
Chicken ownership	Milk	5(9.1%)	5(10.4%)	1.3%	0.851	10(18.2%)	11(22.9%)	4.7%	0.502	3.4%	0.733
	Meat	0.0%	0.0%	0.0%	1.00	4(7.3%)	5(10.4%)	3.1%	0.430	3.1%	0.577
	Egg	8(14.5%)	10(20.8%)	6.3%	0.449	16(29.1%)	35(72.9%)	43.8%	0.000	37.5%	0.002

## Discussion

This study examined the possible effect of a community-based nutrition education on the improvement of ASFs consumption and increased dietary diversity among infants and young children in Northern Ethiopia. It confirmed that nutrition educational intervention delivered through the HEWs in mothers’ home could significantly improve the consumption of ASFs among infants and young children in the intervention group (DID of 19.5, p = 0.008). Although the dietary diversity of the complementary diets remained very low, like in other parts of the country [10, 45], it was marginally significant; considerably higher children in the intervention than comparison group at endline were fed on diverse diets (DID of 9.5, p *=* 0.062). This is in line with previous interventional studies in Malawi [46] and Uganda [18]. Similarly, endline survey in Ethiopia results following the intervention of nutritional education and counseling, showed significant differences in the proportion of minimum dietary diversity between intervention and control groups [26, 27, 47]. These results indicated that an education intervention delivered through local health services could enhance children’s complementary feeding practices and ultimately improve children’s growth.

The proportion of children who received minimum meal frequency (MMF) was low in both areas at baseline. The practice is lower as compared to EDHS report 41.9% [45]. Although there was no significant difference between the intervention and comparison groups, slightly more children reached MMF in the intervention groups during the endline. This finding is supported by a study conducted in other parts of Ethiopia [48], which reported that no significant difference in the improvement of MMF among the study groups after the intervention.

The present study showed that the intervention had significantly influenced the consumption of eggs (p = 0.012) among other ASFs. However, no child was consuming meat during the baseline and even it was very low at endline despite being dense in a wide range of quality protein and micronutrients (such as zinc, iron, and vitamin A) linked to child growth and cognitive development [49]. It is likely that meat consumption increases as infants’ age increase [32]. In line with the current study, earlier reports from Ethiopia revealed that low consumption of meat among infants and young children were widespread [10, 14, 50], which could be explained by the low access and high price hampers its consumption [8, 51, 52]. Thus, economic empowerment and education intervention to mothers about the nutritional values to infants and young children seemed to ensure the consumption of ASFs.

Animal source foods are an essential component of a nutritious diet, which infants and young children rarely consume despite households’ ownership of livestock. Earlier studies in Ethiopia reported that livestock is considered as an indicator of wealth and hence it is seldom consumed [9, 10]. But the current findings indicate that there is an opportunity for livestock owners to provide ASFs for their children. It was found that consumption of eggs was associated with chicken ownership in households after the education intervention. The intervention significantly influenced consumption of eggs (p = 0.002) which was increased by 37.5 percentage points. Similarly, research findings in Nepal [23], Rural Tanzania [53] and Malawi [54] revealed that chicken ownership had increased the consumption of eggs among young children. This could partly explain that eggs can easily be obtained from own product and local market with a reasonable price, and it is easy to prepare. A formative research in Northern Ethiopia revealed that during a card-sorting exercise with a selected group of women and men, who described eggs as the most healthy and acceptable ASF for children, which is easy to prepare, and locally available [55].

Another key finding of the current study indicated that the proportion of children that consumed eggs in the intervention group was higher than the comparison group in households that owned sheep and goats. Indeed, keeping livestock couldn’t only provide a regular supply of nutrient-rich ASFs; selling self-produced animals or animal products can increase household income that could be used to purchase micro-nutrient-rich foods [52]. Thus, the present finding could result indirectly through purchasing from income generated after selling sheep and goats; the pathway needs further research.

Although many researchers reported that cow ownership enhances milk consumption by children [56, 57], no statistically significant association between cow ownership and child milk consumption was observed in the current study. A proposed reason for this finding might be due to the farmers having poor breed cows that could give birth at an interval of four years or more [8]. Besides, households are unwilling to sell or slaughter their cattle because cattle are considered prestigious, even in the face of infertility [58]. However, further investigations are needed.

Similarly, there was no significant change in milk consumption in the intervention compared to the comparison area of sheep and goats ownership, although the intervention has contributed to some of the changes in sheep and goats ownership. This finding aligns with previous studies, which found that in rural Ethiopia, a women-focused goat development program positively affected young children’s milk consumption [4]. These results suggest that there may be room to improve the extent to which livestock ownership could provide benefits to consume ASFs among children. Hence, developing awareness about the importance of ASF to children should be addressed through appropriate nutrition education.

### Limitations

The study has some limitations. The study relied on mothers’ ability to recall child food intake accurately; although the questionnaire was pre-tested, and enumerator training was in-depth. Food insecurity of households is widespread in the study area, affecting the consumption of ASFs among children. Moreover, the study didn’t account for seasonal variations during the intervention time. But both the intervention and comparison arms are food insecure and have exposed similar seasonal variations. The poor breeds of cows, time and labour inputs with livestock rearing activities didn’t consider which could affect mothers’ child feeding practices [54]. The indirect consumption pathways were not considered, and the mechanisms by which the intervention might have an effect were not discussed.

### Conclusions

Although ASFs provide critical micronutrients deficient in children’s diets, its consumption among infants and young children is very low in the study area. The present findings suggest that simply owning livestock does not directly improve ASFs consumption among children. But livestock ownership positively influenced the consumption of ASFs by children after nutrition education interventions. Thus, the study has contributed to the evidence about the encouraging role of nutrition education interventions focusing on the consumption of ASFs from their livestock to overcome the low consumption of ASFs to improve the quality and diversity of complementary diets. It also suggests that the need for involvement of other family members using similar approaches, who would provide a supportive setting where livestock ownership turns into actual consumption of ASFs for children. Interventions that promote the pathway between household livestock ownership and child dietary intake could provide nutritional benefits among rural children.

## Supporting information

S1 Table Intervention activities and nutrition education sessions provided to health extension workers and mother-child pairs. (DOCX)

S2 Table Baseline assessment of children with 6–8 months age who consumed different food groups across each Tabias of the intervention and comparison groups. (DOCX)

S1 Questionnaire (DOCX)

S1 File Baseline vs endline data set. (DTA)
