# Supplementary material for: Educational intervention and livestock ownership successfully improved the intake of animal source foods in 6–23 months old children in rural communities of Northern Ethiopia: Quasi-experimental study
Source: PLoS One. 2022 Nov 4;17(11):e0277240. doi: 10.1371/journal.pone.0277240 (PMC9635712; doi:10.1371/journal.pone.0277240)
Supplement: S1 Table — (DOCX) [file pone.0277240.s001.docx]

**S1 File:** Table 1 Intervention activities and nutrition education sessions provided to health extension workers and mother-child pairs

| Activities | Topic | Intervention components |
| --- | --- | --- |
| Training of six HEWs and three supervisors from the health center of the study district by researchers | Introduction | - Intervention objective, content and activities of intervention, prevalence of child under-nutrition in Tigray, why nutrition matters during the first two years of life, breastfeeding, feeding principles and practices, feeding frequency, and food groups, the benefits of ASFs for 6-23 months old children. - Training resource materials such as module, picture and leaflet |
|  | Barriers and facilitators for consumption of ASFs among 6-23 months old children | - Discussing the barriers and available resources for optimal consumption of ASFs among children in the study area based on the qualitative findings and the proposed solutions listed below. |
| -Follow-up visits to mothers at home (by HEWs)  -Each HEW will be assigned 25 mothers | - **Barriers to consumption of ASFs among children:** - Lack of nutrition knowledge - High cost of ASFs - Low household income - Livestock products as sources of cash/income - Social norms and beliefs (fasting time, no teeth to chew meat, served first to the father) | - ASFs help children to gain weight, good health, grows strong and active. Provide it every day. - ASFs have good nutritive benefits (such as protein, iron, zinc, calcium, vitamin A, vitamin B12) for child growth and mental development) - Cook, mash, and feed ASFs (e.g. eggs, beef, mutton, chicken) - Provide whole milk and undiluted milk rather than the skim milk - Cook and boil rather than serving in the raw form - Keep ASFs in a hygienic way to prevent any zoonotic and foodborne diseases - During selling of ASFs, advise mothers to purchase other food groups to children (eg. Fruits and vegetables, less price animal products) - Priority is given for infants and young children rather than others (fathers) - Discuss the beliefs and norms that affect the consumption of ASFs among children and mothers should encourage the consumption of ASFs during the religious fasting season - Discuss the role of ASFs in promoting growth and good health, and cereal-based complementary foods have incomplete protein and micronutrients - Reduce the market-oriented production of ASFs and share part of the products for their child consumption - Enrich complementary foods by adding ASFs such as milk and egg during preparation of porridge made from a combination of cereal flours. - Feed sick baby during and after illness (diversity, frequency, consistency, and amount). - Hygiene (safety, handling & storage of food), home and environment sanitation - Allocate a separate feeding plate to quantify food for child |
|  | - Facilitators of ASFs consumption - Livestock ownership - Livestock in-kind credit programs - Availability of nutrition expert - Cooking demonstrations | - Nutrition experts promote the consumption of ASFs among infants and young children - Cooking demonstrations and picture-based training on the importance of ASFs and child development - Counsel mothers to provide ASFs from their own production (e.g. if they have cow milk, chicken, sheep and/goat, the mothers will be counseled to provide milk, egg or chicken meat) - Enrichment of the complementary food with dry meat (quanta) powder by purchasing fresh meat from butchery shop |
| Supervisory visits | Supervisory visits to HEWs and mothers. Feedback regarding the set goals will be reviewed. | Education evaluation and review of home visits through checking for individual counseling based on the contents of the nutrition education sessions provided to mothers. Such as:   1. Check whether mother X (name of the mother) is visited by HEWs every other week? 2. Check if mother X own any livestock such as cow, sheep, goat, chicken 3. If mother X owned livestock, for what purpose the livestock product she used? (such as household consumption, sale, gift, others) 4. If it is for household consumption (Q_3_), what ASFs mother X provided to her child (egg, meat, milk, others) 5. If it is for sale (Q_3_), what other products mother X purchased to her child? (Fruits and vegetables, less price other animal products, others) 6. Did mother X provide ASFs for her child during your visit? (yes, no) 7. If yes (Q_6_), how she provided meat to her child? (dry and mash it, same as other families, not provide, others) 8. If yes (Q_6_), how she provided milk to her child? (whole milk, diluted with water, skim milk, not provide, others) 9. If no (Q_6_), how often to fed her child with ASFs? (every other day, weekly, no need of it, holiday, others) 10. Did mother X know the benefit of ASFs for her child? (yes, no) 11. If yes (Q_10_), what she knew about benefit of ASFs to children? (Gain weight, good health, grows strong and active, others). 12. Did mother X feed her child ASFs in raw form? (yes, no) 13. If no (Q_12_), why? (fear of zoonotic disease, not easily chew, culture, others) 14. Did mother X allocate a separate feeding plate to her child (yes, no) 15. For whom ASFs were providing priority in the household? (mothers, fathers, children, adolescents, others) 16. Did mother X provide ASFs to her child during fasting day? (yes, no) 17. If no (Q16), why? (fear of contamination to other family, children should fast, no need of ASFs to children, lack of ASFs, others) 18. What ASFs mother X added to her child’s complementary foods such as porridge made from a combination of cereal flours? (milk, egg, meat, no, others) 19. How mother X kept ASFs in a hygienic way? (proper handling and storage, lack of attention, the same as other food items, others) 20. Did you face any challenges during your counseling? (yes, no) 21. If yes (Q_20_), what was and how did you solve it? |
| Meeting | A three to four hour joint meeting among the principal investigator, supervisors and HEWs | - How education and counseling sessions and taught lessons would hold with mothers? - Number of home visits carried out by HEWs and number of mothers attending each education and counseling session - Number dropped out and its reason - Observation of mothers’ attentiveness and interest (e.g. asked/answered questions) - Do mothers are provided cooking demonstration of ASFs - Do children consume ASFs - Discussing any challenges faced during the intervention period and its solution |

Table2. Schedule of intervention activities during the study period

| **Activities** | **Time point** | **Frequency** | **Target group** | **Conducted by** |
| --- | --- | --- | --- | --- |
| Training of community-based nutrition counsellors and supervisors | Two days | Once at outset | Health facility staff and HEWs | Research team |
| Education and counselling of mothers through home visits | Eighteen times | Every two weeks. Mothers with sick children or children with feeding problems will be offered additional visits. | Study subjects | HEWs |
| Supervision of community-based nutrition counsellors | Nine times | Every months | HEWs and study subjects | Health facility staff (supervisors) |
| Performance review and joint discussion | Nine times | Every month. But, phone communication- at least weekly | Researchers, health facility staff and HEWs | Researcher |
| Data collection | Two times | At the beginning before the intervention and one month after the last educational sessions | Study subjects | trained data collectors |

Note: data were collected from both the intervention and comparison groups of the study area.
