# Supplementary material for: Educational intervention and livestock ownership successfully improved the intake of animal source foods in 6–23 months old children in rural communities of Northern Ethiopia: Quasi-experimental study
Source: PLoS One. 2022 Nov 4;17(11):e0277240. doi: 10.1371/journal.pone.0277240 (PMC9635712; doi:10.1371/journal.pone.0277240)
Supplement: S2 Table — (DOCX) [file pone.0277240.s002.docx]

**S3 File**: Table 2. Baseline assessment of children with 6-8 months age who consumed different food groups across each Tabias of the intervention and comparison groups

(Jijke=49; Simret=69; T/weini=35 and Adikaela=46; Adishishay= 44; Mariam Moko=50)

| Food groups | Comparison  Tabias | Consumption (%) | (P-value) | Intervention Tabias | Consumption (%) | (P-value) |
| --- | --- | --- | --- | --- | --- | --- |
| Grains | Jijke | 42(85.7) | 0.778 | Adikaela | 37(80.4) | 0.758 |
|  | Simret | 58(84) |  | Adishishay | 37(84.1) |  |
|  | T/weini | 28(80) |  | Mariam Moko | 43(86.0) |  |
| Legumes and nuts | Jijke | 20(40.8) | 0.807 | Adikaela | 15(32.6) | 0.089 |
|  | Simret | 25(36.2) |  | Adishishay | 24(54.6) |  |
|  | T/weini | 12(34.3) |  | Mariam Moko | 19(38.0) |  |
| Dairy products | Jijke | 8(16.3) | 0.113 | Adikaela | 6(13.0) | 0.372 |
|  | Simret | 6(8.7) |  | Adishishay | 2(4.6) |  |
|  | T/weini | 1(2.9) |  | Mariam Moko | 5(10.0) |  |
| Meat | Jijke | 0 | 1.00 | Adikaela | 0 | 1.00 |
|  | Simret | 0 |  | Adishishay | 0 |  |
|  | T/weini | 0 |  | Mariam Moko | 0 |  |
| Eggs | Jijke | 7(14.3) | 0.143 | Adikaela | 9(19.6) | 0.322 |
|  | Simret | 11(15.9) |  | Adishishay | 4(9.1) |  |
|  | T/weini | 1(2.9) |  | Mariam Moko | 6(12.0) |  |
| Vitamin A-rich fruits and vegetables | Jijke | 1(2.1) | 0.954 | Adikaela | 1(2.2) | 0.270 |
|  | Simret | 2(2.9) |  | Adishishay | 1(2.3) |  |
|  | T/weini | 1(2.9) |  | Mariam Moko | 4(8.0) |  |
| Other fruits and vegetables | Jijke | 7(14.3) | 0.233 | Adikaela | 9(19.6) | 0.868 |
|  | Simret | 4(5.8) |  | Adishishay | 7(15.9) |  |
|  | T/weini | 5(14.3) |  | Mariam Moko | 8(16.0) |  |
| Minimum diet diversity | Jijke | 3(6.1) | 0.367 | Adikaela | 1(2.2) | 0.553 |
|  | Simret | 1(1.5) |  | Adishishay | 3(6.8) |  |
|  | T/weini | 1(2.9) |  | Mariam Moko | 3(6.0) |  |
| Minimum meal frequency | Jijke | 18(36.7) | 0.214 | Adikaela | 15(32.6) | 0.358 |
|  | Simret | 18(26.1) |  | Adishishay | 10(22.7) |  |
|  | T/weini | 7(20.0) |  | Mariam Moko | 18(36.0) |  |
