# Supplementary material for: Educational intervention and livestock ownership successfully improved the intake of animal source foods in 6–23 months old children in rural communities of Northern Ethiopia: Quasi-experimental study
Source: PLoS One. 2022 Nov 4;17(11):e0277240. doi: 10.1371/journal.pone.0277240 (PMC9635712; doi:10.1371/journal.pone.0277240)
Supplement: S1 Questionnaire — (DOCX) [file pone.0277240.s003.docx]

**S2 file: Questionnaire**

**Date_______________**

**1. Characteristics of the study children and respondents**

| RN | Question | Response |
| --- | --- | --- |
| 1.1 | **Study area** | |
| 1 | Region |  |
| 2 | Zone |  |
| 3 | Woreda |  |
| 4 | Tabia |  |
| 5 | Kushet |  |
| 1.2 | **Household information** | |
| 1 | Sex of child | Male, female |
| 2 | Age of child (day/ months/year) |  |
| 3 | Number of children < five years in the household | One, two, three and above |
| 4 | Child birth order | First, second, third, fourth, fifth |
| 5 | Sex head household | Male, female |
| 6 | Age head household |  |
| 7 | Religion head household | Orthodox, Muslim, protestant, others |
| 8 | Number of family in the household |  |
| 9 | Educational attainment of mothers | No formal education, Primary education, High school, College education, Bachelor degree and above |
| 10 | Educational attainment of fathers | No formal education, Primary education, High school, College education, Bachelor degree and above |
| 11 | Occupational status of mothers & fathers |  |

**2. Characteristics of house and assets**

| RN | **Characteristics of house and assets** | | | |
| --- | --- | --- | --- | --- |
| 1 | What is the main material of the roof? | 1 = thatch/grass or leaves  2 = iron sheets  3 = other…specify | |  |
| 2 | What is the main material of the walls? | 1 = no walls  2 = natural materials cane, wood, mud  3 = planks  4 = stone with mud  5 = stone with cement/bricks  6 = other | |  |
| 3 | What is the main floor material? | 1 = natural floor (earth/sand/dung)  2 = rudimentary floor (wood/palm/bamboo)  3 = finished floor (polished wood, vinyl, tiles, cement, carpet)  4 = other | |  |
| 4 | What kind of toilet facilities does your household have? | 1= no facility/bush/field  2= pit toilet/latrine  3= Ventilated improved pit latrine  4= flush toilet  5=other | |  |
| 5 | What is the main source of drinking water for members of your household? | 1=piped water into dwelling  2=piped water into compound  3=public tap  4=borehole in compound  5=dug well in compound  6=water from protected spring  7=tanker truck  8=surface water (river/dam/lake etc.)  09=bottled water  10=unprotected spring  88=other | |  |
| 6 | Do you do anything to the water to make it safer to drink? | 1 = Yes  2 = No  3 = Don’t know | |  |
| 7 | If yes Q no 6, what is the main thing you do? | 1 = Let it stand and settle  2 = Strain through a cloth  3 = Use water filter (ceramic/sand/composite/etc.)  4 = Boil  5 = Solar disinfection  6 = Add bleach/chlorine  7 = Other  8 = Don’t know | |  |
| 8 | What type of fuel does your household mainly use for cooking? | 1=electricity  2=gas  3=kerosene  4=charcoal  5=firewood/straw  6=dung  7=other | |  |
| 9 | Is the house connected to electricity? | 1=yes  2=no | |  |
| 10 | In total, how many of the following items are owned by residents of this household?  *Add the household total for each item* |  | *Enter number of items (zero if none)* | |
|  |  | 10. 1 | Wrist watch |  |
|  |  | 10. 2 | Gold (in grams) |  |
|  |  | 10. 3 | A kerosene lamp/pressure lamp |  |
|  |  | 10. 4 | Solar lamp |  |
|  |  | 10. 5 | A bed |  |
|  |  | 10. 6 | Non-mobile phone |  |
|  |  | 10. 7 | Mobile phone |  |
|  |  | 10. 8 | Bicycle |  |
|  |  | 10. 9 | Car |  |
|  |  | 10. 10 | Radio |  |
|  |  | 10. 11 | TV |  |
|  |  | 10. 12 | Fridge |  |
|  |  | 10. 13 | Motor bike |  |
|  |  | 10. 14 | Bajaj |  |
| 11 | Do you own this house? | 1 = yes  2 = no | |  |
| 12 | Does any member of the household own any agricultural land? | 1 = yes  2 = no | |  |
| 13 | If yes for Q12, how many hectares of agricultural land do members of this household own? | *Enter total number of hectares*  *(If less than 1, Enter in decimals (example 0.5)*  *Enter 9999 if hectares are not known* | |  |

**3. Livestock ownership patterns and their incomes**

3.1. Does this household own any livestock, herds, other farm animals, or poultry?

| 3.2. Does this household own any livestock, herds, other farm animals, or poultry? | 1 = yes  2 = no | | |
| --- | --- | --- | --- |
| 3.3. If yes for Q3.2, how many of the following animals do this household own? | *For each: Enter number. If none, enter 000* | | |
|  | 1 | Chickens |  |
|  | 2 | Goats |  |
|  | 3 | Sheep |  |
|  | 4 | Donkeys |  |
|  | 5 | Horses |  |
|  | 6 | Mules |  |
|  | 7 | Camels |  |
|  | 8 | Milk cows |  |
|  | 9 | Ox (Bulls) |  |
|  | 10 | Others |  |

3.4. If you own livestock, for what purpose do you use?

a) Meat b) milk c) egg d) traction power e) drought power f) sale g) others

3.5. What is the purpose of ASFs? (a) Household consumption (b) sale (c) others

3.6. Does livestock ownership increase the likelihood of consuming other high value foods? (yes, no), if yes: a) Cereals b) pulses c) leafy vegetables d) vegetables e) fruits f) others

3.7. Which animal type has great income contribution to the household?

a) Cattle b) sheep and goat c) chicken d) bee e) drought animals

3.8. What is the price of livestock food? (per kg); (a) beef ____(b) goat meat____ (c) sheep meat ____ (d) milk ____ (e) chicken _____

**4. Dietary diversity**

| Ask the mothers on breastfeeding status and list all liquids or food consumed by each child yesterday during the day or at night. It is interested in whether the child had the item it is mentioned even if it was combined with other foods Please write down other foods in this box that respondent mentioned but are not in the list below. |
| --- |

| Group | Food lists | Yes | No |
| --- | --- | --- | --- |
| Group 1, Cereals. roots and tubers | enjera, bread, rice, porridge, or other foods made from cereals and roots and tubers (such as Teff, Finger Millet, Rice, maize, wheat, sorghum, potato etc.) |  |  |
| Group 2: Legumes and nuts | Any foods made from legumes and nuts such as beans, chick-peas, peas, lentils, kidney bean, nuts or seeds etc |  |  |
| Group 3: Dairy products | Infant formula, such as [insert local examples] | How many times? |  |
|  | Milk and milk products such as tinned, powdered or fresh animal milk | How many times? |  |
|  | Yogurt or drinking yogurt | How many times? |  |
| Group 4: Flesh foods | Liver, kidney, heart or other organ meats |  |  |
|  | Any meat, such as beef, lamb, goat, chicken |  |  |
| Group 5: Eggs | Eggs |  |  |
| Group 6: Vitamin A rich fruits and vegetables | Pumpkin, carrots, squash or sweet potatoes that are yellow or orange inside etc |  |  |
|  | Any dark green vegetables [insert local examples] |  |  |
|  | Ripe mangoes (fresh or dried [not green]), ripe papayas (fresh or dried), musk melon [insert other local vitamin-A-rich fruits] |  |  |
|  | Foods made with red palm oil, red palm nut or red palm nut pulp sauce |  |  |
| Group 7: Other fruits and vegetables | Any other fruits or vegetables |  |  |
| Others (not counted in the dietary diversity score) | Any oil, fats, or butter or foods made with any of these |  |  |
|  | Any sugary foods, such as chocolates, sweets, candies, pastries, cakes or biscuits |  |  |
|  | Condiments for flavor, such as chilies, spices, herbs or fish powder |  |  |

**5. Animal source foods consumption patterns**

5.1. Do all family members eat animal source foods? (a)Yes (b) no

5.2. If no (Q5.1), which groups of the family members do not eat animal source foods? Why? a) Child b) wife c) husband d) others

___________________________________________________________________________

5.3. If no (Q5.1), which groups of the family members do not take animal source foods/ which animal source foods? (a) beef ____(b) goat meat____ (c) sheep meat ____ (d) milk ____ (e) chicken _____

What are the reasons? (a) high price (b) no access of ASFs (c) fear of zoonotic diseases (d) culturally not eaten (e) others

5.4. If yes for (Q5.1), why do you eat animal source foods? (a) Cheap price (b) good taste (c) have high nutrients (d) medicinal reasons (e) It is our culture (f) own product (g) others

5.5. Do you give ASFs to your children? (a) Yes (b) no

5.6. If yes for (Q5.5) at what time of age you start ASFs?

5.7. Whom do you prioritize ASFs in the household? a) father, b) mother c) infant and young children d) children e) adolescent e) others

**6. Minimum meal frequency**

| 1 | What is the current feeding practice? | 1. only breastfeeding 2. breast milk + animal milk or formula milk 3. c) breastfeeding+ Complementary feeding 4. d) Completely weaned |
| --- | --- | --- |
| 2 | Did (name of the baby) consume breast milk yesterday during the day or at night? | Yes, No, Don’t know/no answer |
|  |  |  |
| 3 | How long is it recommended that a woman breastfeeds her child? | Six months or less, 6–11 months, 12–23 months, 24 months and more, Other, Don’t know |
| 4 | Did (name of the baby) eat any solid, semi-solid, or soft foods yesterday during the day or at night? | Yes, No  If ‘Yes’ What kind of solid, semi-solid or soft foods did eat? (dietary diversity questions) |
| 5 | At what age should babies start eating foods in addition to breast milk? | At six months, Other, Don’t know |
| 6 | How many times did (name of the baby) eat foods that is meals and snacks other than liquids yesterday during the day or at night? | Number of times ________, On demand, whenever the baby wants,  Don’t know/no answer |
